# The Association between *KCNQ1* Gene Polymorphism and Type 2 Diabetes Risk: A Meta-Analysis

**DOI:** 10.1371/journal.pone.0048578

**Published:** 2012-11-02

**Authors:** Qiman Sun, Kang Song, Xizhong Shen, Yu Cai

**Affiliations:** 1 Liver Cancer Institute, Zhongshan Hospital, Fudan University, Shanghai, People’s Republic of China; 2 Department of Gastroenterology, Zhongshan Hospital, Fudan University, Shanghai, People’s Republic of China; Children’s Hospital Boston, United States of America

## Abstract

**Background:**

KCNQ1 (potassium voltage-gated channel KQT-like sub-family, member 1) encodes a pore-forming subunit of a voltage-gated K^+^ channel (KvLQT1) that plays a key role for the repolarization of the cardiac action potential as well as water and salt transport in epithelial tissues. Recently, genome-wide association studies have identified KCNQ1 as a type 2 diabetes (T2D) susceptibility gene in populations of Asian descent. After that, a number of studies reported that the rs2237892 and rs2237895 polymorphism in KCNQ1 has been implicated in T2D risk. However, studies on the association between these polymorphism and T2D remain conflicting. To investigate this inconsistency, we performed this meta-analysis.

**Methods:**

Databases including Pubmed, EMBASE, Web of Science and China National Knowledge Infrastructure (CNKI) were searched to find relevant studies. Odds ratios (ORs) with 95% confidence intervals (CIs) were used to assess the strength of association. Potential sources of heterogeneity were also assessed by subgroup analysis and meta-regression.

**Results:**

A total of 25 articles involving 70,577 T2D cases and 99,068 controls were included. Overall, the summary odds ratio of C allele for T2D was 1.32 (95% CI 1.26–1.38; P<10−5) and 1.24 (95% CI: 1.20–1.29; P<10−5) for KCNQ1 rs2237892 and rs2237895 polymorphisms, respectively. Significant results were also observed using co-dominant, dominant and recessive genetic models. After stratifying by ethnicity, sample size, and diagnostic criteria, significant associations were also obtained.

**Conclusions:**

This meta-analysis suggests that the rs2237892 and rs2237895 polymorphisms in KCNQ1 are associated with elevated type 2 diabetes susceptibility.

## Introduction

Type 2 diabetes (T2D) is a complex metabolic disorder characterized by variable degrees of insulin resistance, impaired insulin secretion and elevated blood glucose. It has become a global major health problem showing worldwide increasing prevalence with an estimated 300 million people predicted to develop the disease by 2025 [1]. Although its exact etiology is unknown, a combination of multiple genetic and environmental factors is considered to contribute to the pathogenesis of the disease. Until recently, few genes identified through the candidate gene approach have been confirmed to be associated with T2D (e.g., PPARG, KCNJ11, CAPN10, and TCF7L2). However, because the pathogenesis of T2D is yet to be elucidated completely, the candidate-gene approach is limited in power to detect novel disease-susceptibility genes.

Recently, spectacular advance was made in identifying susceptible genes involved in T2D through genome-wide association strategy (GWAS) [2]. KCNQ1 (potassium voltage-gated channel KQT-like subfamily, member 1) is a gene encoding the pore-forming subunit of a voltage-gated K^+^ channel (KvLQT1) that plays a key role for the repolarization of the cardiac action potential as well as water and salt transport in epithelial tissues [3]–[5]. Mutations in the KCNQ1 gene cause the long QT syndrome and deafness [6]. In addition, KCNQ1 is also expressed in pancreatic islets and the cultured insulin-secreting INS-1 cells [7], [8], and blockade of the channel with KCNQ1 inhibitors 293B stimulated insulin secretion [8], suggesting that KCNQ1 channels may play a role in regulation of insulin secretion.

Several important single nucleotide polymorphisms (SNPs) have been identified in the *KCNQ1* gene. Two polymorphisms (rs2237892 and rs2237895) which were in moderate linkage disequilibrium (LD) were first identified to be associated with increased T2D risk in Asians through genome wide association approach (r^2^ = 0.30) [9]. The relationship between KCNQ1 polymorphisms and T2D has been reported in various ethnic groups. However, recent studies [10], [11] found no association between the two polymorphisms and T2D. These disparate findings may be due partly to insufficient power, false-positive results, and publication biases. The interpretation of these studies has been further complicated by the use of different populations, or different control source. To help clarify the inconsistent findings, we conducted a comprehensive meta-analysis to quantify the overall risk of KCNQ1 polymorphism on developing T2D.

## Materials and Methods

### Literature Search Strategy

Genetic association studies published before the end of June 2012 on T2D and polymorphisms in the *KCNQ1* gene were identified through a search of PubMed, Web of Science, EMBASE and CNKI (Chinese National Knowledge Infrastructure) with keywords “KQT-like subfamily, member 1”, “*KCNQ1*”, “type 2 diabetes mellitus”, “type 2 diabetes”, “T2D”, “T2DM”. All references cited in these studies and published reviews were examined in order to identify additional work. Eligible studies had to meet all of the following criteria: (a) should have been published in peer-reviewed journal; (b) were independent association studies investigating polymorphism with T2D using original data; (c) should have presented sufficient data to calculate the odds ratio (OR) with confidence interval (CI) and p value, and (d) should have described the genotyping method, equipment, and protocols used or provided reference to them. The major reasons for exclusion of studies were (a) overlapping data, (b) case-only studies and (c) review papers.

### Data Extraction

Data extraction was performed independently by two reviewers and differences were resolved by further discussion among all authors. For each included study, the following information was extracted from each report according to a fixed protocol: first author, publication year, definition and numbers of cases and controls, diagnostic criterion, genotype frequency, source of controls, gender, body mass index (BMI), Hardy–Weinberg equilibrium (HWE) status, ethnicity and genotyping method.

### Statistical Methods

The strength of association between polymorphisms of *KCNQ1* and T2D risk was assessed by odds ratio (OR) with the corresponding 95% confidence interval (CI). The per-allele OR of the risk allele was compared between cases and controls using co-dominant genetic model. Additional pooled estimates were also given with corresponding results under co-dominant, dominant and recessive genetic models. Heterogeneity across individual studies was calculated using the Cochran’s X^2^ based Q-statistic test followed by subsidiary analysis or by random-effects regression models with restricted maximum likelihood estimation [12]–[15]. Random-effects and fixed-effect summary measures were calculated as inverse variance-weighted average of the log OR. The results of random-effects summary were reported in the text because it takes into account the variation between studies. Ethnicity, diagnostic criterion (World Health Organization or American Diabetes Association criterion), study size (≥1000 and <1000 cases) were pre-specified as characteristics for assessment of heterogeneity. Ethnic group was defined as Caucasian (i.e., people of European origin), East Asian, South Asian (i.e., Indian) and others. Ethnicity, BMI, diagnostic criterion, sample size, mean age at test and sex distribution in cases and controls were analyzed as covariates in meta-regression. Funnel plots was used to provide diagnosis of the potential publication bias. Egger’s regression test was also conducted to identify small study effects [15]. Sensitivity analysis, which determines the influence of individual studies on the pooled estimate, was also performed to assess the stability of the result. All P values are two-sided at the P = 0.05 level. All statistical analyses were carried out with the Stata software version 10.0 (Stata Corporation, College Station, TX).

## Results

### Characteristics of Studies

The combined search yielded 85 references. 60 articles were excluded because they clearly did not meet the criteria or overlapping references (Figure S1). A total of 25 studies were finally included with 70,577 T2D cases and 99,068 controls [9]–[11], [16]–[37]. The detailed characteristics of the included studies were shown in Table 1. There are 36 data sets from 22 studies with 63,760 T2D cases and 89,709 controls concerning rs2237892 and 26 data sets from 15 studies involving 37,822 T2D cases and 47,195 controls concerning rs2237895. These two polymorphisms were found to occur in frequencies consistent with Hardy-Weinberg equilibrium in the control populations of the vast majority of the published studies. Of the cases, 69.2% were East Asians, 23.5% were Caucasians, 5.8% were South Asians and 1.5% were of other ethnic origins.

**Table 1:** Characteristics of the studies included in the meta-analysis.

Study	Year	Ethnicity	No. of cases/controls	Diagnostic criteria	Definition of control	Age of cases/controls	Sex distribution in cases/controls(% male)	Genotyping method
Yasuda [9]	2008	Japanese, Chinese, Korean	9182/9959	WHO	Non-diabetic participants	NA/NA	NA/NA	GeneChip, PCR-based Invader assay
Unoki [16]	2008	Japanese	3463/1313	WHO	Normoglycemic	NA/NA	NA/NA	GeneChip
Lee [17]	2008	Korean	865/496	ADA	Normal fasting glucose	58/55	4854	TaqMan
Takeuchi [18]	2009	Japanese	5619/7359	WHO	Non-diabetic participants	63/65	61/48	GeneChip, TaqMan,MassARRAY
Qi [19]	2009	Chinese	424/1908	WHO	Normal fasting glucose	60/58	NA/NA	GenomeLab SNPstream
Jonsson [20]	2009	Swedish	2684/5482	ADA	Normal fasting glucose	58/58	59/38	TaqMan
Hu [21]	2009	Chinese	1719/1720	WHO	Normal glucose tolerence	61/57	52/41	MassARRAY
Liu [22]	2009	Chinese	1885/1994	WHO	Normal fasting glucose	54/58	41/31	TaqMan
Chen [10]	2010	Chinese	57/341	T2D patient	Normal fasting glucose	NA/52	NA/48	TaqMan
Yamauchi [23]	2010	Japanese	7764/6432	WHO	Non-diabetic participants	65/58	NA/NA	GeneChip
Han [24]	2010	Chinese	990/959	WHO	Normal glucose tolerence	56/58	34/53	SNaPshot
Tsai [25]	2010	Chinese	2798/2367	ADA	Non-diabetic participants	60/49	52/50	GeneChip
Tan [26]	2010	Chinese, Malaysian, Indian	4817/2863	WHO	Normal glucose tolerence	NA/NA	NA/NA	MassARRAY
Grallert [27]	2010	German	1186/1405	ADA	Normoglycemic	61/61	55/55	TaqMan
Voight [28]	2010	European	8130/38987	WHO	Non-diabetic participants	NA/NA	NA/NA	GeneChip,TaqMan, KASPar,MassARRAY
Xu [29]	2010	Chinese	1891/2852	WHO	Normal glucose regulation	63/60	44/38	SNaPshot
Zhou [30]	2010	Chinese	537/510	WHO	Normal fasting glucose	57/56	43/37	MassARRAY
Been [11]	2011	Indian	1428/1593	WHO	Normoglycemic	NA/NA	NA/NA	TaqMan
Saif-Ali [31]	2011	Malaysian	234/177	T2D patient	Non-diabetic participants	48/45	45/46	RFLP
Tabara [32]	2011	Japanese	493/394	ADA	Normal glucose tolerence	60/59	55/53	TaqMan
Odgerel [33]	2011	Mongolian	177/216	WHO	Non-diabetic participants	NA/NA	NA/NA	TaqMan
Saif-Ali [34]	2011	Chinese	300/230	T2D patient	Non-diabetic participants	50/53	51/61	RFLP
van Vliet-Ostaptchouk [35]	2012	Dutch	4549/5182	WHO	Healthy participants	64/51	43/42	TaqMan
Campbell [36]	2012	Latin American	876/399	WHO	Non-diabetic participants	63/61	49/NA	SNPlex
Yu [37]	2012	Chinese	8509/3930	WHO	Normal glucose tolerence	NA/NA	48/41	MassARRAY

NA: Not Available, WHO: World Health Organization, ADA: American Diabetes Association.

### Association of rs2237892 Polymorphism with T2D

**Figure 1:**
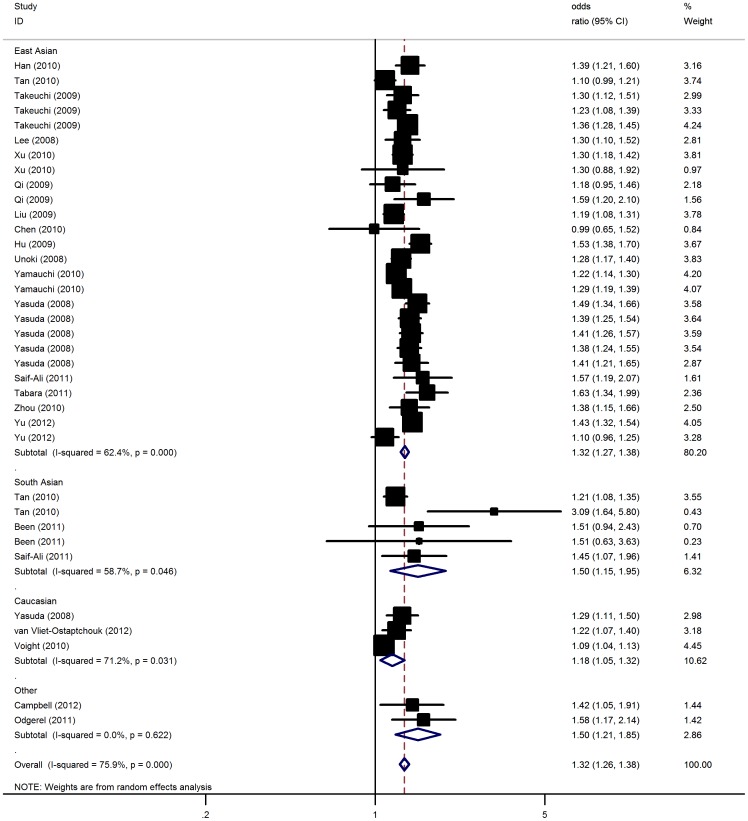
Meta-analysis of the association between KCNQ1 rs2237892 polymorphism and the risk for type 2 diabetes mellitus.

Overall, there was evidence of an association between the increased risk of T2D and the variant in different genetic models when all the eligible studies were pooled into the meta-analysis (Figure 1). Using random effect model, the summary per-allele OR of the C variant for T2D was 1.32 [95% CI: 1.26–1.38, *P*(Z)<10^−5^; *P*(Q)<10^−5^], with corresponding results under dominant and recessive genetic models of 1.62 [95% CI: 1.51–1.74, *P*(Z) <10^−5^; *P*(Q) = 0.0006] and 1.40 [95% CI: 1.33–1.48, *P*(Z)<10^−5^; *P*(Q) <10^−4^], respectively. Similar results were also detected under co-dominant model (Table 2).

**Table 2:** Meta-analysis of the *KCNQ1* rs2237892 polymorphism on type 2 diabetes risk.

Sub-group analysis	No. of data sets	No. of case/control	C allele			Heterozygote			Homozygote			Dominant model			Recessive model		
			OR (95%CI)	P(Z)	P(Q)	OR (95%CI)	P(Z)	P(Q)	OR (95%CI)	P(Z)	P(Q)	OR (95%CI)	P(Z)	P(Q)	OR (95%CI)	P(Z)	P(Q)
Overall	36	63760/89709	1.32 (1.26–1.38)	<10^−5^	<10^−5^	1.41 (1.31–1.52)	<10^−5^	0.11	1.86 (1.71–2.02)	<10^−5^	0.04	1.62 (1.51–1.74)	<10^−5^	0.0006	1.40 (1.33–1.48)	<10^−5^	<10^−4^
Ethnicity																	
East Asian	26	43023/38454	1.32 (1.27–1.38)	<10^−5^	<10^−5^	1.42 (1.33–1.52)	<10^−5^	0.24	1.88 (1.74–2.03)	<10^−5^	0.08	1.63 (1.53–1.74)	<10^−5^	0.002	1.41 (1.33–1.50)	<10^−5^	<10^−4^
South Asian	5	4284/3075	1.50 (1.15–1.95)	0.002	0.05	1.60 (1.07–2.12)	0.008	0.09	1.72 (1.14–2.23)	<10^−4^	0.58	1.41 (1.20–1.68)	0.005	0.03	1.30 (1.18–1.44)	0.0007	0.82
Caucasian	3	15400/47565	1.18 (1.05–1.32)	0.006	0.03	1.13 (1.03–1.23)	0.003	0.04	1.27 (1.09–1.30)	0.01	0.15	1.20 (1.06–1.36)	0.007	0.15	1.28 (1.16–1.42)	<10^−5^	0.27
Others	2	1053/615	1.50 (1.21–1.85)	0.0001	0.62	1.36 (1.10–1.64)	0.006	0.05	1.46 (1.19–1.97)	0.0003	0.21	1.17 (1.07–1.28)	0.02	0.18	1.11 (1.04–1.19)	0.01	0.07
Sample size																	
Small	16	6793/9180	1.40 (1.31–1.49)	<10^−5^	0.29	1.49 (1.25–1.79)	<10^−4^	0.23	2.00 (1.71–2.33)	<10^−5^	0.40	1.73 (1.47–2.03)	<10^−5^	0.003	1.44 (1.32–1.58)	<10^−5^	0.005
Large	20	56967/80529	1.28 (1.22–1.35)	<10^−5^	<10^−5^	1.39 (1.28–1.51)	<10^−5^	0.12	1.82 (1.65–2.01)	<10^−5^	0.02	1.59 (1.48–1.72)	<10^−5^	0.008	1.39 (1.29–1.49)	<10^−5^	<10^−4^
Diagnostic criterion																	
WHO criterion	30	59052/84645	1.31 (1.25–1.37)	<10^−5^	<10^−5^	1.43 (1.31–1.56)	<10^−5^	0.03	1.87 (1.70–2.03)	<10^−5^	0.02	1.62 (1.50–1.76)	<10^−5^	0.009	1.39 (1.31–1.48)	<10^−5^	0.0001
ADA criterion	3	4117/4316	1.38 (1.20–1.59)	<10^−4^	0.13	1.34 (1.04–1.74)	0.02	0.59	2.00 (1.41–2.82)	<10^−4^	0.23	1.62 (1.25–2.11)	0.0003	0.34	1.46 (1.21–1.77)	<10^−4^	0.09
HWE status																	
Yes	34	57987/88849	1.32 (1.27–1.38)	<10^−5^	<10^−5^	1.40 (1.30–1.52)	<10^−5^	0.10	1.88 (1.73–2.05)	<10^−5^	0.05	1.63 (1.51–1.75)	<10^−5^	0.007	1.43 (1.36–1.49)	<10^−5^	0.002
No	2	5773/860	1.75 (0.64–4.81)	0.27	0.002	1.68 (0.75–3.92)	0.31	0.01	1.86 (0.59–4.97)	0.19	0.007	1.55 (0.94–2.17)	0.18	0.01	1.01 (0.86–1.20)	0.89	0.009

P(Z): Z test used to determine the significance of the overall OR.

P(Q): Cochran’s chi-square Q statistic test used to assess the heterogeneity in subgroups.

When studies were stratified for ethnicity, significantly increased risks were found among East Asian populations (C allele: OR = 1.32, 95% CI: 1.27–1.38; dominant model: OR = 1.63, 95% CI: 1.53–1.74; recessive model: OR = 1.40, 95% CI: 1.33–1.48), and South Asian (C allele: OR = 1.50, 95% CI: 1.15–1.95; dominant model: OR = 1.41, 95% CI: 1.20–1.68; recessive model: OR = 1.30, 95% CI: 1.18–1.44). Similar significant associations were also observed for Caucasians (C allele: OR = 1.18, 95% CI: 1.05–1.32; dominant model: OR = 1.20, 95% CI: 1.06–1.36; recessive model: OR = 1.28, 95% CI: 1.16–1.42). By considering sample size subgroups, the OR was 1.28 (95% CI: 1.22–1.35; *P*<10^−5^) in large studies compared to 1.40 (95% CI: 1.31–1.49; *P*<10^−5^) in small studies. In the stratified analysis by diagnostic criterion, significant associations were found for WHO criterion with per-allele OR of 1.31 (95% CI: 1.25–1.37; P<10^−5^) and for ADA criterion of 1.38 (95% CI: 1.20–1.59; P<10^−4^). Subsidiary analyses of HWE status yielded a per-allele OR for controls consistent to HWE of 1.32 (95% CI: 1.27–1.38), while no significant results were detected for studies deviated from HWE. Similar results were also found using co-dominant, dominant or recessive genetic model (Table 2). After adjusting for multiple testing using Bonferroni correction, all significant associations for rs2237892 under the co-dominant, dominant and recessive genetic models remained.

Significant heterogeneity was present among the 36 data sets (P<0.05). In meta-regression analysis, diagnostic criterion (*P* = 0.39), mean age of cases (*P* = 0.11) and controls (*P* = 0.51), mean BMI of case (*P* = 0.65) and controls (*P* = 0.39), sex distribution in cases (*P* = 0.30) and controls (P = 0.38) did not significantly explained such heterogeneity. By contrast, ethnicity (*P* = 0.03) and sample size (*P* = 0.03) were significantly correlated with the magnitude of the genetic effect.

### Association of rs2237895 Polymorphism with T2D

**Figure 2:**
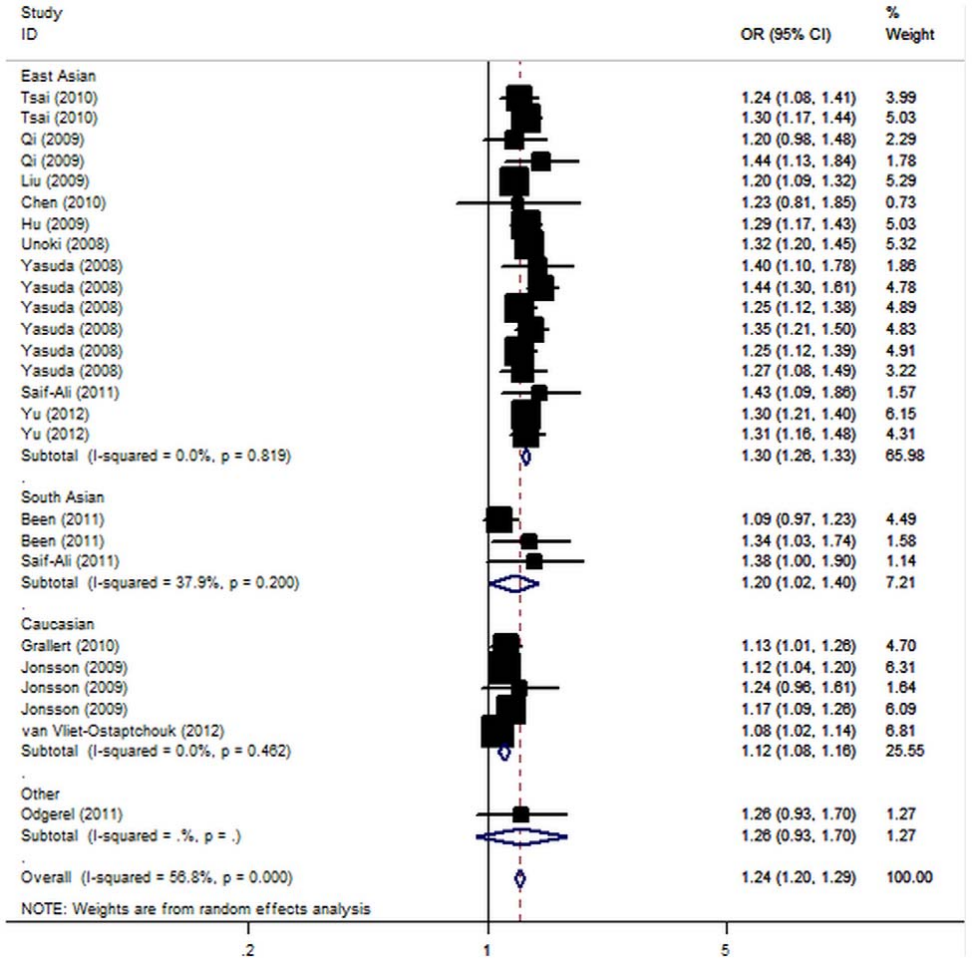
Meta-analysis of the association between KCNQ1 rs2237895 polymorphism and the risk for type 2 diabetes mellitus.

For T2D risk and the rs2237895 polymorphism of *KCNQ1*, our meta-analysis gave an overall OR of 1.24 (95% CI: 1.20–1.29; *P*<10^−5^; Figure 2) with statistically significant between-study heterogeneity (P<10^−5^). Significantly increased T2D risks were also found under dominant [OR = 1.32, 95% CI: 1.24–1.39, *P*(Z) <10^−5^; *P*(Q) <10^−4^] and recessive [OR = 1.35, 95% CI: 1.26–1.45; *P*(Z) <10^−5^; *P*(Q) = 0.001] genetic model. Significant associations were also found using co-dominant model (Table 3).

**Table 3:** Meta-analysis of the *KCNQ1* rs2237895 polymorphism on type 2 diabetes risk.

Sub-group analysis	No. of data sets	No. of case/control	C allele			Heterozygote			Homozygote			Dominant model			Recessive model		
			OR (95%CI)	P(Z)	P(Q)	OR (95%CI)	P(Z)	P(Q)	OR (95%CI)	P(Z)	P(Q)	OR (95%CI)	P(Z)	P(Q)	OR (95%CI)	P(Z)	P(Q)
Overall	26	37822/47195	1.24 (1.20–1.29)	<10^−5^	<10^−5^	1.25 (1.19–1.32)	<10^−5^	0.009	1.54 (1.41–1.68)	<10^−5^	0.0004	1.32 (1.24–1.39)	<10^−5^	<10^−4^	1.35 (1.26–1.45)	<10^−5^	0.001
Ethnicity																	
East Asian	17	25713/20820	1.30 (1.26–1.33)	<10^−5^	0.82	1.30 (1.24–1.36)	<10^−5^	0.68	1.69 (1.57–1.81)	<10^−5^	0.85	1.37 (1.32–1.44)	<10^−5^	0.57	1.47 (1.38–1.57)	<10^−5^	0.65
South Asian	3	1658/1759	1.20 (1.02–1.40)	0.03	0.20	1.18 (1.01–1.38)	0.04	0.64	1.42 (1.01–2.01)	0.03	0.19	1.21 (1.05–1.41)	0.01	0.41	1.25 (1.01–1.64)	0.04	0.26
Caucasian	5	10274/24400	1.12 (1.08–1.16)	<10^−5^	0.46	1.16 (1.02–1.33)	0.01	0.007	1.24 (1.15–1.34)	<10^−5^	0.65	1.18 (1.05–1.33)	0.002	0.07	1.19 (1.10–1.27)	<10^−4^	0.49
Others	1	876/399	1.26 (0.93–1.70)	0.13	NA	1.14 (0.98–1.33)	0.08	NA	1.22 (0.97–1.55)	0.10	NA	1.08 (0.97–1.20)	0.15	NA	1.14 (0.98–1.33)	0.08	NA
Sample size																	
Small	11	3382/7910	1.29 (1.21–1.38)	<10^−5^	0.98	1.31 (1.14–1.50)	0.0001	0.95	1.74 (1.42–2.14)	<10^−5^	0.94	1.40 (1.23–1.59)	<10^−5^	0.83	1.51 (1.26–1.81)	<10^−4^	0.61
Large	15	34440/39285	1.23 (1.18–1.29)	<10^−5^	<10^−5^	1.25 (1.17–1.33)	<10^−5^	0.0007	1.51 (1.37–1.67)	<10^−5^	<10^−4^	1.31 (1.22–1.40)	<10^−5^	<10^−4^	1.33 (1.23–1.44)	<10^−5^	0.003
Diagnostic criterion																	
WHO criterion	17	28708/24862	1.26 (1.20–1.33)	<10^−5^	<10^−5^	1.26 (1.17–1.34)	<10^−5^	0.002	1.59 (1.43–1.76)	<10^−5^	0.0004	1.33 (1.24–1.42)	<10^−5^	0.0001	1.40 (1.29–1.51)	<10^−5^	0.006
ADA criterion	6	8523/21585	1.18 (1.12–1.24)	<10^−5^	0.24	1.23 (1.11–1.36)	0.0001	0.25	1.29 (1.16–1.42)	<10^−5^	0.70	1.24 (1.14–1.36)	<10^−5^	0.31	1.22 (1.07–1.35)	<10^−5^	0.22

NA: Not Available.

P(Z): Z test used to determine the significance of the overall OR.

P(Q): Cochran’s chi-square Q statistic test used to assess the heterogeneity in subgroups.

When stratifying for ethnicity, significant risks were found among East Asians in all genetic model (C allele: OR = 1.30, 95% CI: 1.26–1.33; dominant model: OR = 1.37, 95% CI: 1.32–1.44; recessive model: OR = 1.47, 95% 1.38–1.57). Similar results were also found in the Caucasian populations (C allele: OR = 1.12, 95% CI: 1.08–1.16; dominant model: OR = 1.18, 95% CI: 1.05–1.33; recessive model: OR = 1.19, 95% CI: 1.10–1.27). Among South Asian populations, only marginally significant results were detected (C allele: OR = 1.20, 95% CI: 1.02–1.40; dominant model: OR = 1.21, 95% CI: 1.05–1.41; recessive model: OR = 1.25, 95% CI: 1.01–1.64). In considering sample size subgroups, the OR was 1.29 (95% CI: 1.21–1.38; P<10^−5^) in small studies compared to 1.23 (95% CI: 1.18–1.29; P<10^−5^) in large studies. Further stratified according diagnostic criterion, significant results were found for both WHO and ADA criterion in all genetic models (Table 3). After adjusting for multiple testing using Bonferroni correction, all significant associations for rs2237895 under the co-dominant, dominant and recessive genetic models remained.

In meta-regression analysis, neither sample size (*P* = 0.24), diagnostic criterion (*P* = 0.50), mean BMI of cases (*P* = 0.37) and controls (*P* = 0.26), mean age of cases (*P* = 0.05) and controls (*P* = 0.22), nor sex distribution in cases (*P* = 0.64) and controls (*P* = 0.61) were significantly correlated with the magnitude of the genetic effect; while ethnicity (P = 0.02) explained a large part of the heterogeneity.

### Sensitivity Analyses and Publication Bias

A single study involved in the meta-analysis was deleted each time to reflect the influence of the individual data-set to the pooled ORs, and the corresponding pooled ORs were not qualitatively altered. The shape of the funnel plots was symmetrical for these polymorphisms (Figure S2 and S3). The statistical results still did not show small study effects in these studies for rs2237892 (Egger test, *P* = 0.12) and rs2237895 (Egger test, *P* = 0.07).

## Discussion

Multiple lines of evidence support an important role for genetics in determining risk for T2D. Two independent GWA studies recently performed in Japanese populations identified KCNQ1 as a T2D susceptibility gene [9], [16]. After that, a number of studies reported that common SNPs in KCNQ1 have been implicated in T2D risk. However, studies on the association between these polymorphism and T2D remain conflicting. This is the first meta-analysis involving a total of 169,645 subjects from 25 case-control studies examining the association of two commonly studied polymorphisms (rs2237892 and rs2237895) of KCNQ1 with T2D risk.

Our results demonstrated that the C alleles of rs2237892 and rs2237895 polymorphism of KCNQ1 are a risk factor for developing T2D. In the stratified analysis by ethnicity, significant associations were observed among different populations in all genetic models among East Asian, Caucasian and South Asian populations, which suggested a similar role of the polymorphism in different ethnicity with different genetic backgrounds and living environment. Meta-analysis is often dominated by a few large studies, which markedly reduces the evidence from smaller studies. By considering sample size, significantly increased T2D susceptibility in KCNQ1 risk allele carriers was also found both in large and small studies for all genetic models. However, our results suggest an overestimation of the true genetic association by small studies. Besides, studies using different diagnostic criterion also get consistent positive results. Furthermore, we explored potential sources of heterogeneity across studies and the possibility of publication bias.

The K_V_-channels are believed to play an important role in the pancreatic β-cells mediating repolarization of the membrane terminating Ca^2+^-influx and insulin secretion, and a K_V_-channel knock-out in rat islets as well as pharmacological inhibition of K_V_-channels in mouse β-cells have been reported to enhance glucose-stimulated insulin secretion [38]–[41]. The K_V_ 7.1 channel, encoded by KCNQ1, is expressed in INS-1 cells and has been suggested to play an important role in maintaining the membrane potential in these cells [8]. Based on in vitro data and the association with T2D, there is compelling evidence suggesting an effect also on T2D-related quantitative traits with variation in this gene. The analyzed polymorphism rs2237892 was found to be associated with a fasting parameter of insulin secretion [homoestasis model assessment of β-cell function (HOMA-B)] in a Japanese sample and with an oral glucose tolerance test-derived insulin secretion parameter (corrected insulin response) in a European sample [9]. In addition, Qi et al. also confirmed the association of the KCNQ1 variants with impaired β-cell function estimated by HOMA-B [19]. Moreover, Jonsson et al. conducted a prospective population based study and found that rs2237895 indeed increases risk of future T2D and that this is due to failing β-cell function [20]. Recently, a variant in the KCNQ1 gene (rs2237892) was reported to be associated with second-phase insulin secretion by hyperglycaemic clamp technique [35]. This observation indicates that KCNQ1 might play a role in second-phase insulin secretion suggesting a novel potential link between KCNQ1 and impaired β-cell function via the decreased release of newly formed insulin following glucose stimulation. These association and functional studies combined with our meta-analysis suggest the KCNQ1 variants may be implicated in the pathogenesis of T2D mainly through impaired insulin secretion of pancreatic β-cells. Since rs2237892 and rs2237895 is located near the outside of an KCNQ1 exon, it does not change the amino acid sequence, indicating that further study of the biological function of this SNP is necessary.

Although it has been known for decades that both type 2 diabetes and obesity have a genetic basis [42], only a few of risk genes with robust and reproducible effects have been identified for these diseases. Unfortunately, almost all the studies included in current meta-analysis did not explore the interaction between KCNQ1 genotype and obesity. Recently, Yu et al. found that rs2237892 was associated with waist circumference, but the significance was not retained after adjusting for body mass index (BMI), which implied that KCNQ1 was most likely involved in overall adiposity but not central adiposity [37]. In addition, the risk alleles for T2D at KCNQ1 (C alleles of rs2237892 and rs2237895) were associated with a reduced risk of being overweight and obese as well as a decreased BMI in diabetic individuals [21], [37]. Regarding the interaction between rs2237892 and BMI on the risk of type 2 diabetes, and the finding that the effect of rs2237892 on diabetes risk was greater for individuals with lower BMI than for those with higher BMI, it is hypothesized that KCNQ1 might participate in the pathogenesis of T2D via non-BMI-mediated pathways [37].

Several potential limitations of the present meta-analysis should be taken into consideration. First, although the funnel plot and Egger’s test showed no publication bias and although an exhaustive literature search was done, it is likely that some publications and unpublished data were overlooked. Selection bias for the meta-analysis might have occurred. Second, our results were based on unadjusted estimates, whereas a more precise analysis could be conducted if all individual raw data were available, which would allow for the adjustment by other co-variables including age, drinking status, obesity, cigarette consumption, and other lifestyle factors. Third, lack of individual-level data prevent us from making further analysis to identify any interactions between genetic variation and metabolic traits (fasting plasma glucose, indices for insulin sensitivity, or beta-cell function).

In summary, this meta-analysis showed that the KCNQ1 rs2237892 and rs2237895 polymorphism was significantly associated with increased risk of T2D. As studies among Middle East and African populations are currently limited, further studies including a wider spectrum of subjects to investigate the role of this locus in these populations will be needed. Moreover, gene–gene and gene–environment interactions should also be considered in future studies.

## Supporting Information

Figure S1 **The flow chart of the included studies.**
(TIF)

Figure S2 **Begg’s funnel plot of KCNQ1 rs2237892 polymorphism and type 2 diabetes.**
(TIF)

Figure S3 **Begg’s funnel plot of KCNQ1 rs2237895 polymorphism and type 2 diabetes.**
(TIF)

Checklist S1 (DOC)

## References

[1] van Tilburg J, van Haeften TW, Pearson P, Wijmenga C (2001) Defining the genetic contribution of type 2 diabetes mellitus. J Med Genet 38: 569–578. 11546824 10.1136/jmg.38.9.569 PMC1734947

[2] Frayling TM (2007) Genome-wide association studies provide new insights into type 2 diabetes aetiology. Nat Rev Genet 8: 657–662. 17703236 10.1038/nrg2178

[3] Barhanin J, Lesage F, Guillemare E, Fink M, Lazdunski M, et al (1996) K(V)LQT1 and IsK (minK) proteins associate to form the I(Ks) cardiac potassium current. Nature 384: 78–80. 8900282 10.1038/384078a0

[4] Wang Q, Curran ME, Splawski I, Burn TC, Millholland JM, et al (1996) Positional cloning of a novel potassium channel gene: KVLQT1 mutations cause cardiac arrhythmias. Nat Genet 12: 17–23. 8528244 10.1038/ng0196-17

[5] Neyroud N, Tesson F, Denjoy I, Leibovici M, Donger C, et al (1997) A novel mutation in the potassium channel gene KVLQT1 causes the Jervell and Lange-Nielsen cardioauditory syndrome. Nat Genet 15: 186–189. 9020846 10.1038/ng0297-186

[6] Splawski I, Shen J, Timothy KW, Lehmann MH, Priori S, et al (2000) Spectrum of mutations in long-QT syndrome genes: KVLQT1, HERG, SCN5A, KCNE1, and KCNE2. Circulation 102: 1178–1185. 10973849 10.1161/01.cir.102.10.1178

[7] Demolombe S, Franco D, de Boer P, Kuperschmidt S, Roden D, et al (2001) Differential expression of KvLQT1 and its regulator IsK in mouse epithelia. Am J Physiol Cell Physiol 280: C359–C372. 11208532 10.1152/ajpcell.2001.280.2.C359

[8] Ullrich S, Su J, Ranta F, Wittekindt O, Ris F, et al (2005) Effects of IKs channel inhibitors in insulin-secreting INS-1 cells. Pflugers Arch 451: 428–436. 16133261 10.1007/s00424-005-1479-2

[9] Yasuda K, Miyake K, Horikawa Y, Hara K, Osawa H, et al (2008) Variants in KCNQ1 are associated with susceptibility to type 2 diabetes mellitus. Nat Genet 40: 1092–1097. 18711367 10.1038/ng.207

[10] Chen Z, Zhang X, Ma G, Qian Q, Yao Y (2010) Association study of four variants in KCNQ1 with type 2 diabetes mellitus and premature coronary artery disease in a Chinese population. Mol Biol Rep 37: 207–212. 19575309 10.1007/s11033-009-9597-0

[11] Been LF, Ralhan S, Wander GS, Mehra NK, Singh J, et al (2011) Variants in KCNQ1 increase type II diabetes susceptibility in South Asians: a study of 3,310 subjects from India and the US. BMC Med Genet 12: 18. 21261977 10.1186/1471-2350-12-18 PMC3037841

[12] Cochran WG (1954) The combination of estimates from different experiments. Biometrics 10: 101–129.

[13] DerSimonian R, Laird N (1896) Meta-analysis in clinical trials. Control Clin Trials 7: 177–188. 10.1016/0197-2456(86)90046-2 3802833

[14] Thompson SG, Sharp SJ (1999) Explaining heterogeneity in meta-analysis: a comparison of methods. Stat Med 18: 2693–2708. 10521860 10.1002/(sici)1097-0258(19991030)18:20<2693::aid-sim235>3.0.co;2-v

[15] Egger M, Davey Smith G, Schneider M, Minder C (1997) Bias in meta-analysis detected by a simple, graphical test. BMJ 315: 629–634. 9310563 10.1136/bmj.315.7109.629 PMC2127453

[16] Unoki H, Takahashi A, Kawaguchi T, Hara K, Horikoshi M, et al (2008) SNPs in KCNQ1 are associated with susceptibility to type 2 diabetes in East Asian and European populations. Nat Genet 40: 1098–1102. 18711366 10.1038/ng.208

[17] Lee YH, Kang ES, Kim SH, Han SJ, Kim CH, et al (2008) Association between polymorphisms in SLC30A8, HHEX, CDKN2A/B, IGF2BP2, FTO, WFS1, CDKAL1, KCNQ1 and type 2 diabetes in the Korean population. J Hum Genet 53: 991–998. 18991055 10.1007/s10038-008-0341-8

[18] Takeuchi F, Serizawa M, Yamamoto K, Fujisawa T, Nakashima E, et al (2009) Confirmation of multiple risk Loci and genetic impacts by a genome-wide association study of type 2 diabetes in the Japanese population. Diabetes 58: 1690–1699. 19401414 10.2337/db08-1494 PMC2699880

[19] Qi Q, Li H, Loos RJ, Liu C, Wu Y, et al (2009) Common variants in KCNQ1 are associated with type 2 diabetes and impaired fasting glucose in a Chinese Han population. Hum Mol Genet 18: 3508–3515. 19556355 10.1093/hmg/ddp294

[20] Jonsson A, Isomaa B, Tuomi T, Taneera J, Salehi A, et al (2009) A variant in the KCNQ1 gene predicts future type 2 diabetes and mediates impaired insulin secretion. Diabetes 58: 2409–2413. 19584308 10.2337/db09-0246 PMC2750226

[21] Hu C, Wang C, Zhang R, Ma X, Wang J, et al (2009) Variations in KCNQ1 are associated with type 2 diabetes and beta cell function in a Chinese population. Diabetologia 52: 1322–1325. 19308350 10.1007/s00125-009-1335-6

[22] Liu Y, Zhou DZ, Zhang D, Chen Z, Zhao T, et al (2009) Variants in KCNQ1 are associated with susceptibility to type 2 diabetes in the population of mainland China. Diabetologia 52: 1315–1321. 19448982 10.1007/s00125-009-1375-y PMC2688614

[23] Yamauchi T, Hara K, Maeda S, Yasuda K, Takahashi A, et al (2010) A genome-wide association study in the Japanese population identifies susceptibility loci for type 2 diabetes at UBE2E2 and C2CD4A-C2CD4B. Nat Genet 42: 864–868. 20818381 10.1038/ng.660

[24] Han X, Luo Y, Ren Q, Zhang X, Wang F, et al (2010) Implication of genetic variants near SLC30A8, HHEX, CDKAL1, CDKN2A/B, IGF2BP2, FTO, TCF2, KCNQ1, and WFS1 in type 2 diabetes in a Chinese population. BMC Med Genet 11: 81. 20509872 10.1186/1471-2350-11-81 PMC2896346

[25] Tsai FJ, Yang CF, Chen CC, Chuang LM, Lu CH, et al (2010) A genome-wide association study identifies susceptibility variants for type 2 diabetes in Han Chinese. PLoS Genet 6: e1000847. 20174558 10.1371/journal.pgen.1000847 PMC2824763

[26] Tan JT, Ng DP, Nurbaya S, Ye S, Lim XL, et al (2010) Polymorphisms identified through genome-wide association studies and their ass ociations with type 2 diabetes in Chinese, Malays, and Asian-Indians in Singapore. J Clin Endocrinol Metab 95: 390–397. 19892838 10.1210/jc.2009-0688

[27] Grallert H, Herder C, Marzi C, Meisinger C, Wichmann HE, et al (2010) Association of genetic variation in KCNQ1 with type 2 diabetes in the KORA surveys. Horm Metab Res 42: 149–151. 19798621 10.1055/s-0029-1241170

[28] Voight BF, Scott LJ, Steinthorsdottir V, Morris AP, Dina C, et al (2010) Twelve type 2 diabetes susceptibility loci identified through large-scale association analysis. Nat Genet 42: 579–589. 20581827 10.1038/ng.609 PMC3080658

[29] Xu M, Bi Y, Xu Y, Yu B, Huang Y, et al (2010) Combined effects of 19 common variations on type 2 diabetes in Chinese: results from two community-based studies. PLoS One 5: e14022. 21103332 10.1371/journal.pone.0014022 PMC2984434

[30] Zhou JB, Yang JK, Zhao L, Xin Z (2010) Variants in KCNQ1, AP3S1, MAN2A1, and ALDH7A1 and the risk of type 2 diabetes in the Chinese Northern Han population: a case-control study and meta-analysis. Med Sci Monit 16: BR179–183. 20512086

[31] Saif-Ali R, Muniandy S, Al-Hamodi Z, Lee CS, Ahmed KA, et al (2011) KCNQ1 variants associate with type 2 diabetes in Malaysian Malay subjects. Ann Acad Med Singapore 40: 488–492. 22206064

[32] Tabara Y, Osawa H, Kawamoto R, Onuma H, Shimizu I, et al (2011) Genotype risk score of common susceptible variants for prediction of type 2 diabetes mellitus in Japanese: the Shimanami Health Promoting Program (J-SHIPP study). Development of type 2 diabetes mellitus and genotype risk score. Metabolism 60: 1634–1640. 21550079 10.1016/j.metabol.2011.03.014

[33] Odgerel Z, Lee HS, Erdenebileg N, Gandbold S, Luvsanjamba M, et al (2012) Genetic variants in potassium channels are associated with type 2 diabetes in Mongolian population. J Diabetes 4: 238–242. 22151254 10.1111/j.1753-0407.2011.00177.x PMC3309067

[34] Saif-Ali R, Ismail IS, Al-Hamodi Z, Al-Mekhlafi HM, Siang LC, et al (2011) KCNQ1 Haplotypes Associate with Type 2 Diabetes in Malaysian Chinese Subjects. Int J Mol Sci 12: 5705–5718. 22016621 10.3390/ijms12095705 PMC3189745

[35] van Vliet-Ostaptchouk JV, van Haeften TW, Landman GW, Reiling E, Kleefstra N, et al (2012) Common variants in the type 2 diabetes KCNQ1 gene are associated with impairments in insulin secretion during hyperglycaemic glucose clamp. PLoS One 7: e32148.36. 22403629 10.1371/journal.pone.0032148 PMC3293880

[36] Campbell DD, Parra MV, Duque C, Gallego N, Franco L, et al (2012) Amerind ancestry, socioeconomic status and the genetics of type 2 diabetes in a colombian population. PLoS One 7: e33570. 22529894 10.1371/journal.pone.0033570 PMC3328483

[37] Yu W, Ma RC, Hu C, et al (2012) Association between KCNQ1 genetic variants and obesity in Chinese patients with type 2 diabetes. Diabetologia 55: 2655–2659. 22790062 10.1007/s00125-012-2636-8

[38] MacDonald PE, Ha XF, Wang J, Smukler SR, Sun AM, et al (2001) Members of the Kv1 and Kv2 Voltage-Dependent K+ Channel Families Regulate Insulin Secretion. Mol Endocrinol 15: 1423–1435. 11463864 10.1210/mend.15.8.0685

[39] MacDonald PE, Sewing S, Wang J, Joseph JW, Smukler SR, et al (2002) Inhibition of Kv2.1 Voltage-dependent K+ Channels in Pancreatic beta-Cells Enhances Glucose-dependent Insulin Secretion. J Biol Chem 277: 44938–44945. 12270920 10.1074/jbc.M205532200

[40] Roe MWm, Worley JF III, Mittal AA, Kuznetsov A, DasGupta S, et al (1996) Expression and Function of Pancreatic beta-Cell Delayed Rectifier K+ Channels. Role in Stimulus-Secretion Coupling. J Biol Chem 271: 32241–32246. 8943282 10.1074/jbc.271.50.32241

[41] Zhang M, Houamed K, Kupershmidt S, Roden D, Satin LS (2005) Pharmacological Properties and Functional Role of Kslow Current in Mouse Pancreatic {beta}-Cells: SK Channels Contribute to Kslow Tail Current and Modulate Insulin Secretion. J Gen Physiol 126: 353–363. 16186562 10.1085/jgp.200509312 PMC2266621

[42] Baier LJ, Hanson RL (2004) Genetic studies of the etiology of type 2 diabetes in Pima Indians: hunting for pieces to a complicated puzzle. Diabetes 53: 1181–1186. 15111484 10.2337/diabetes.53.5.1181

