# Supplementary figures and images for: The Association between KCNQ1 Gene Polymorphism and Type 2 Diabetes Risk: A Meta-Analysis
Source: PLoS One. 2012 Nov 2;7(11):e48578. doi: 10.1371/journal.pone.0048578 (PMC3487731; doi:10.1371/journal.pone.0048578)

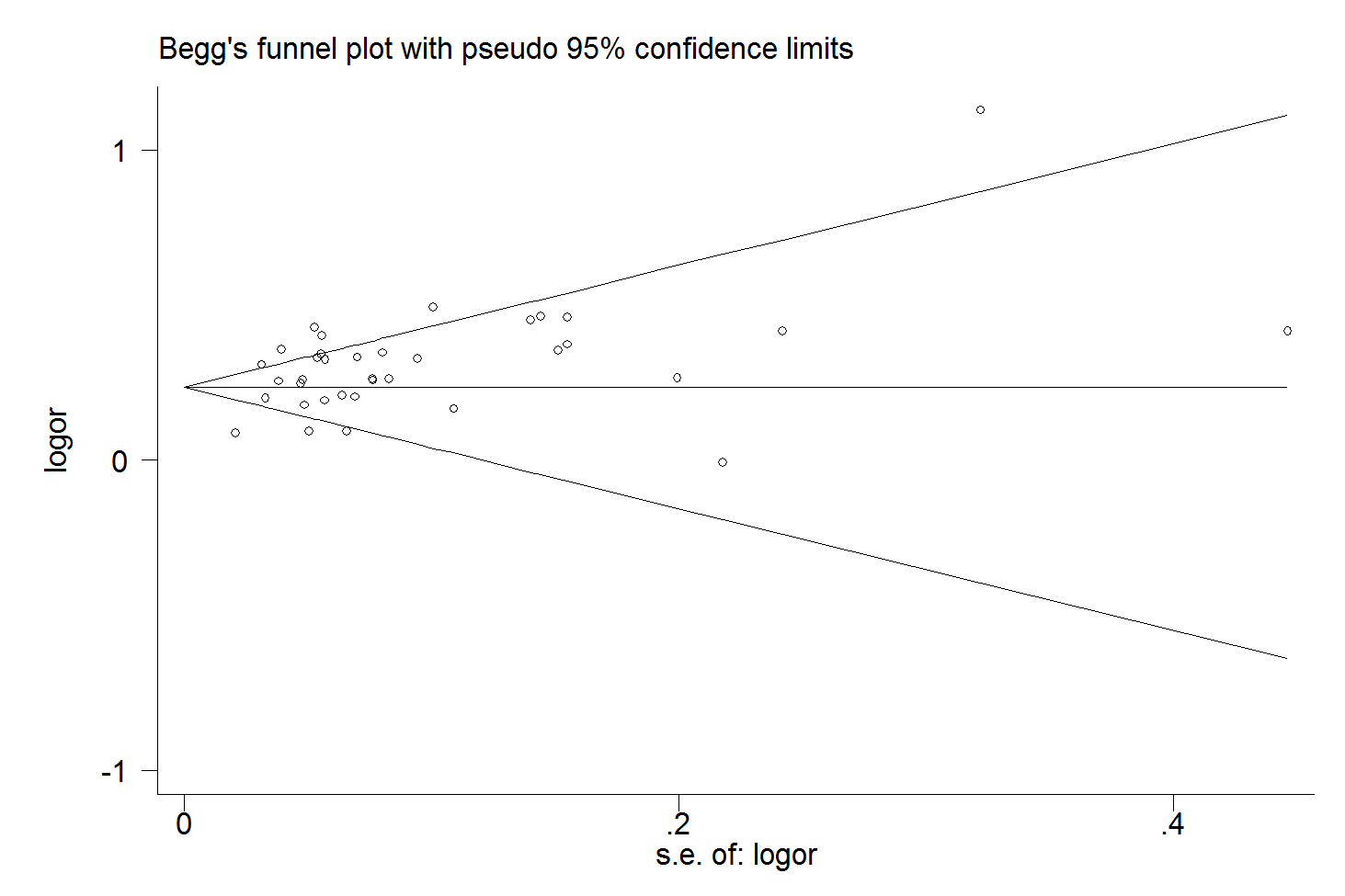

Supplement: Figure S2 — Begg’s funnel plot of KCNQ1 rs2237892 polymorphism and type 2 diabetes. (TIF) [file pone.0048578.s002.tif]

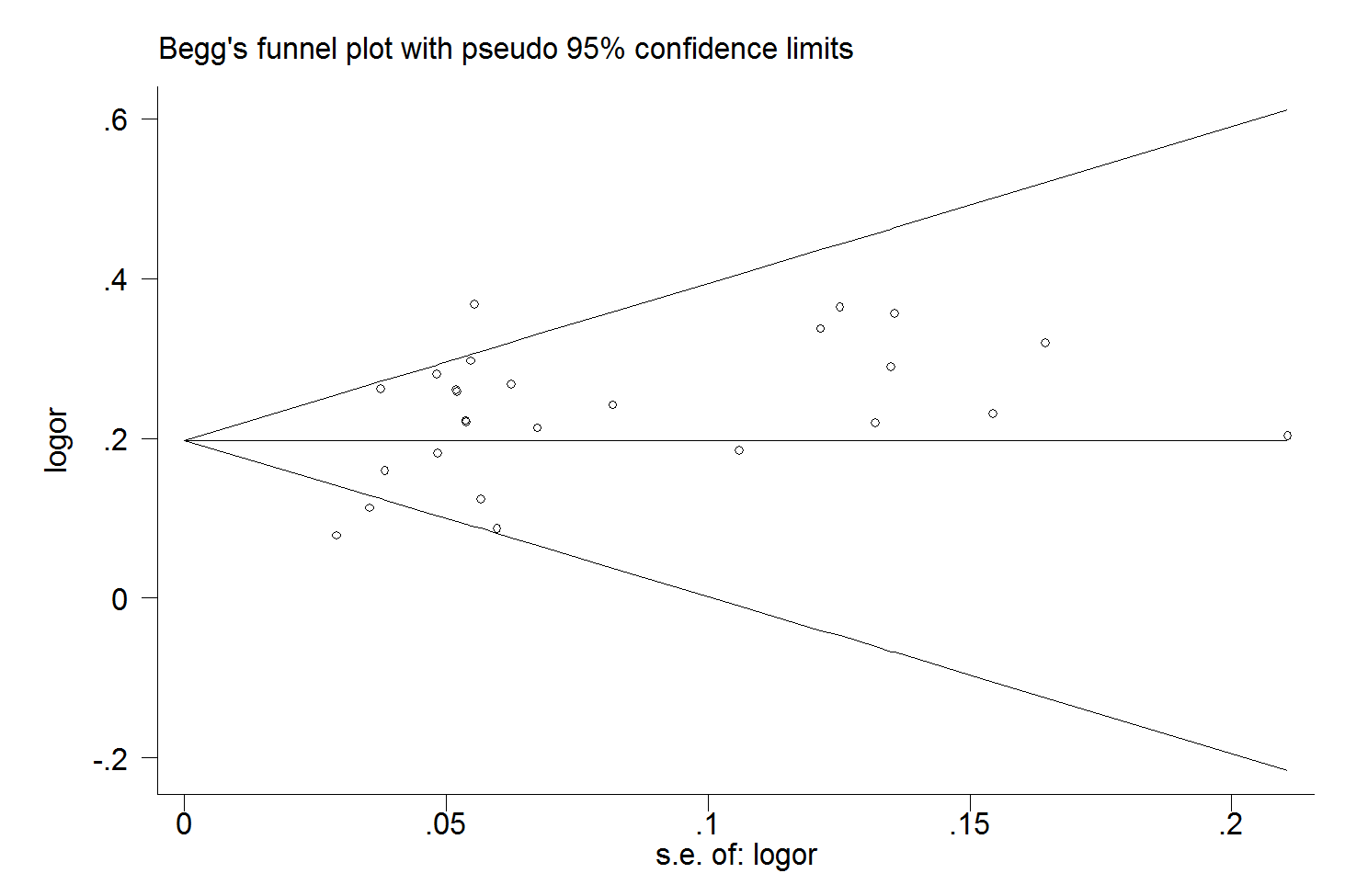

Supplement: Figure S3 — Begg’s funnel plot of KCNQ1 rs2237895 polymorphism and type 2 diabetes. (TIF) [file pone.0048578.s003.tif]
